# Polysulfide evokes acute pain through the activation of nociceptive TRPA1 in mouse sensory neurons

**DOI:** 10.1186/s12990-015-0023-4

**Published:** 2015-05-02

**Authors:** Yukari Hatakeyama, Kenji Takahashi, Makoto Tominaga, Hideo Kimura, Toshio Ohta

**Affiliations:** Department of Veterinary Pharmacology, Faculty of Agriculture, Tottori University, Tottori, 680-8553 Japan; Division of Cell Signaling, Okazaki Institute for Integrative Bioscience (National Institute for Physiological Sciences), National Institutes of Natural Sciences, Okazaki, 444-8787 Japan; Natinal Institute of Neuroscience, National Center of Neurology and Psychiatry, Kodaira, 187-8551 Japan

**Keywords:** Transient Receptor Potential Channels (TRP Channels), Calcium imaging, Dorsal root ganglia, Heterologous expression

## Abstract

**Background:**

Hydrogen sulfide (H_2_S) is oxidized to polysulfide. Recent reports show that this sulfur compound modulates various biological functions. We have reported that H_2_S is involved in inflammatory pain in mice. On the other hand, little is known about the functional role of polysulfide in sensory neurons. Here we show that polysulfide selectively stimulates nociceptive TRPA1 and evokes acute pain, using TRPA1-gene deficient mice (TRPA1(−/−)), a heterologous expression system and a TRPA1-expressing cell line.

**Results:**

In wild-type mouse sensory neurons, polysulfide elevated the intracellular Ca concentration ([Ca^2+^]_i_) in a dose-dependent manner. The half maximal effective concentration (EC_50_) of polysulfide was less than one-tenth that of H_2_S. The [Ca^2+^]_i_ responses to polysulfide were observed in neurons responsive to TRPA1 agonist and were inhibited by blockers of TRPA1 but not of TRPV1. Polysulfide failed to evoke [Ca^2+^]_i_ increases in neurons from TRPA1(−/−) mice. In RIN-14B cells, constitutively expressing rat TRPA1, polysulfide evoked [Ca^2+^]_i_ increases with the same EC_50_ value as in sensory neurons. Heterologously expressed mouse TRPA1 was activated by polysulfide and that was suppressed by dithiothreitol. Analyses of the TRPA1 mutant channel revealed that cysteine residues located in the internal domain were related to the sensitivity to polysulfide. Intraplantar injection of polysulfide into the mouse hind paw induced acute pain and edema which were significantly less than in TRPA1(−/−) mice.

**Conclusions:**

The present data suggest that polysulfide functions as pronociceptive substance through the activation of TRPA1 in sensory neurons. Since the potency of polysulfide is higher than parental H_2_S and this sulfur compound is generated under pathophysiological conditions, it is suggested that polysulfide acts as endogenous ligand for TRPA1. Therefore, TRPA1 may be a promising therapeutic target for endogenous sulfur compound-related algesic action.

## Background

Hydrogen sulfide (H_2_S) is considered to be an endogenous gasotransmitter and is synthesized in the peripheral and central nervous systems [1]. H_2_S exerts various physiological functions through protein sulfhydration [2,3]. It has been reported that H_2_S evokes neurogenic inflammation and hyperalgesia through the activation of various channels, such as transient receptor potential vanilloid 1 (TRPV1) and T-type Ca^2+^ channels [4-7]. We recently reported that H_2_S stimulated a subset of mouse sensory neurons and induced pain-related behaviors [8,9].

TRPA1 and TRPV1 are nonselective cation channels expressed in nociceptive neurons and in part coexpressed in sensory neurons [10]. The TRPA1 channel is activated by a range of natural products [11,12], environmental irritants (acrolein, formalin) [13,14], reactive oxygen species including oxygen [15,16] and cold temperature [17,18]. TRPV1 is also activated by various stimuli such as capsaicin, protons, and noxious heat [19,20]. These channels contribute to the perception of noxious stimuli and play an important role in sensory transduction [21]. They are thought to be associated with inflammatory pain as evidenced in TRPA1 and TRPV1 gene knockout mice [22,23].

Polysulfide, a mixture of substances with varying numbers of sulfurs (H_2_S_n_), is generated from H_2_S in the presence of oxygen [24]. Polysulfide contains sulfane sulfar, which is sustained in various proteins as a potential intracellular H_2_S store to release H_2_S under reduced conditions [25]. It has also been reported that polysulfide is enzymatically biosynthesized by reaction with cysteine [26]. Polysulfide rather than H_2_S has been suggested to be chemical entity to sulfhydrate proteins [27]. The physiological distribution and functions of polysulfide are not well understood. It has recently been reported that polysulfide is found in the brain and activates astrocytes through stimulation of TRPA1, suggesting that it acts as a signaling molecule in the brain [28]. Moreover, polysulfide promotes oxidization of lipid phosphatase and tensin homolog [27]. Though putatively parental H_2_S plays a role in nociception [8], the functional significance of polysulfide in sensory mechanisms and whether polysulfide evokes acute pain are not known.

In the present study, we investigated the effects of polysulfide on sensory neurons in vitro and on nociceptive behavior in vivo using wild-type, TRPV1-null (TRPV1[−/−]), and TRPA1-null (TRPA1[−/−]) mice. To examine the neuronal activity, we used fura-2-based [Ca^2+^]_i_-imaging techniques since most of TRP channels are highly Ca^2+^ permeable [29]. We investigated the effects of polysulfide on cultured mouse dorsal root ganglion (DRG) neurons, which are a useful model of nociception in vitro [8,30,31]. We also used a heterologous expression system to analyze the effects of polysulfide at the molecular level. In addition, we examined whether polysulfide induced acute pain in vivo. The present results indicate that polysulfide excites mouse sensory neurons via the activation of TRPA1 and causes acute pain. Analyses of the TRPA1 mutant channel reveal that cysteine residues located in the N-terminal internal domain are related to the sensitivity to polysulfide.

## Results

### [Ca^2+^]_i_ responses to polysulfide in mouse DRG neurons

**Figure 1:**
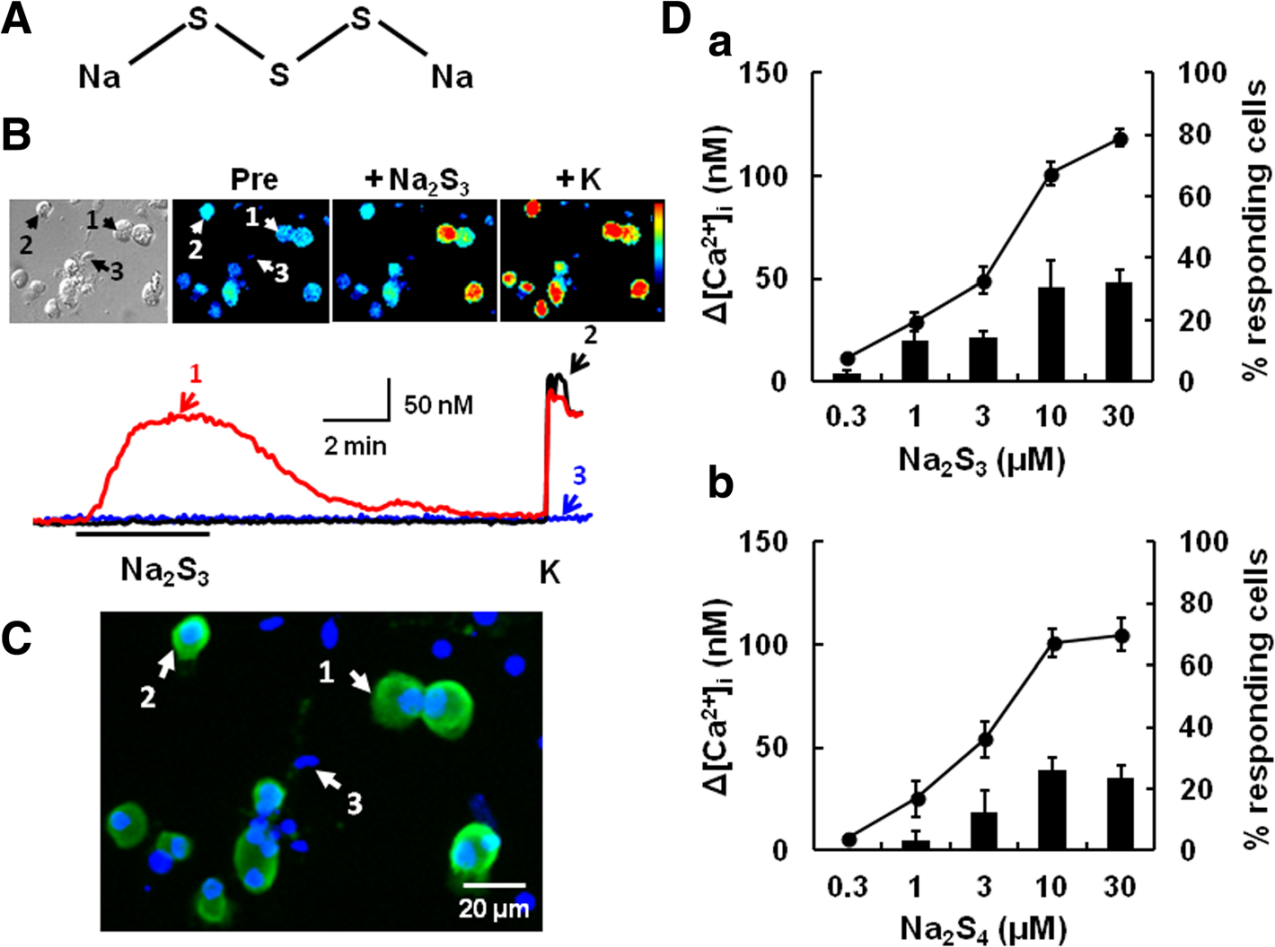
Polysulfide stimulates a subset of mouse sensory neurons. **(A)** The structural formula of Na_2_S_3_. **(B)** An image under transmitted light, and pseudocolor images; before (Pre), after the application of Na_2_S_3_ (+Na_2_S_3,_ 10 μM) and KCl (+K, 80 mM). **(C)** A merged image of immunostaining with antibody against PGP9.5, a neural marker and of nuclear staining with Hoechst 33752. After [Ca^2+^]_i_ responses were measured, cells were subjected to immunostaining.. In (B), cells with arrows (1–3) correspond to cells in the actual recordings and immunocytochemical image. Note that only K-responding cells show positive immunoreactivity to PGP9.5 **(D)** Circles and columns show the concentration-response curve for polysulfide-induced [Ca^2+^]_i_ increases and the percentage of polysulfide-responding neurons among all neurons, respectively (a:Na_2_S_3_, b:Na_2_S_4_). The percentages of polysulfide-responding cells were calculated from the percentage obtained with each coverslip. Symbols with vertical lines show mean ± SEM (Na_2_S_3_; n = 42–74, Na_2_S_4_; n = 33–44, from 3 mice).

Since polysulfide contains a mixture of polymers of different lengths, in the present study we used sodium salts of polysulfide; Na_2_S_3_ (Figure 1A), and Na_2_S_4_. Using the Ca-sensitive dye fura-2, we examined the effects of these polysulfides on changes in the intracellular Ca concentration ([Ca^2+^]_i_) in mouse DRG cells. Actual traces of [Ca^2+^]_i_ and pesudocolor images showed that Na_2_S_3_ (10 μM) elicited [Ca^2+^]_i_ increases in some cells responding to 80 mM KCl (Figure 1B). Since we used 1-day cultured DRG cells (see Methods), it was easy to discriminate neurons from non-neural cells with their size and shape. In a similar morphological and functional way, DRG neurons have been distinguished from non-neural cells [32]. Moreover, KCl-responding cells were immunostained with a neural marker protein gene product 9.5 (PGP9.5) (Figure 1C). [Ca^2+^]_i_ responses to polysulfide peaked during their application, then returned to the basal level. Similar [Ca^2+^]_i_ responses were evoked by Na_2_S_4_. The magnitude of the polysulfide-induced [Ca^2+^]_i_ increases and the percentage of polysulfide-responsive neurons increased in a concentration-dependent manner (Figure 1D). Approximately 30% of the DRG neurons were responsive to both polysulfides at 10 μM or more. It has been reported that bound sulfane sulfurs, including polysulfide, release H_2_S in the presence of reducing agents [24]. We estimated that the H_2_S concentration of 10 μM polysulfide-containing solution was 0.4 μM or less. The EC_50_ values of the two polysulfides were almost the same (4.4 ± 0.17 μM for Na_2_S_3_, 3.9 ± 0.11 μM for Na_2_S_4_). In the following experiments, we used Na_2_S_3_ as polysulfide.

### Polysulfide increases [Ca^2+^]_i_ in mouse DRG neurons sensitive to TRPA1 agonist

**Figure 2:**
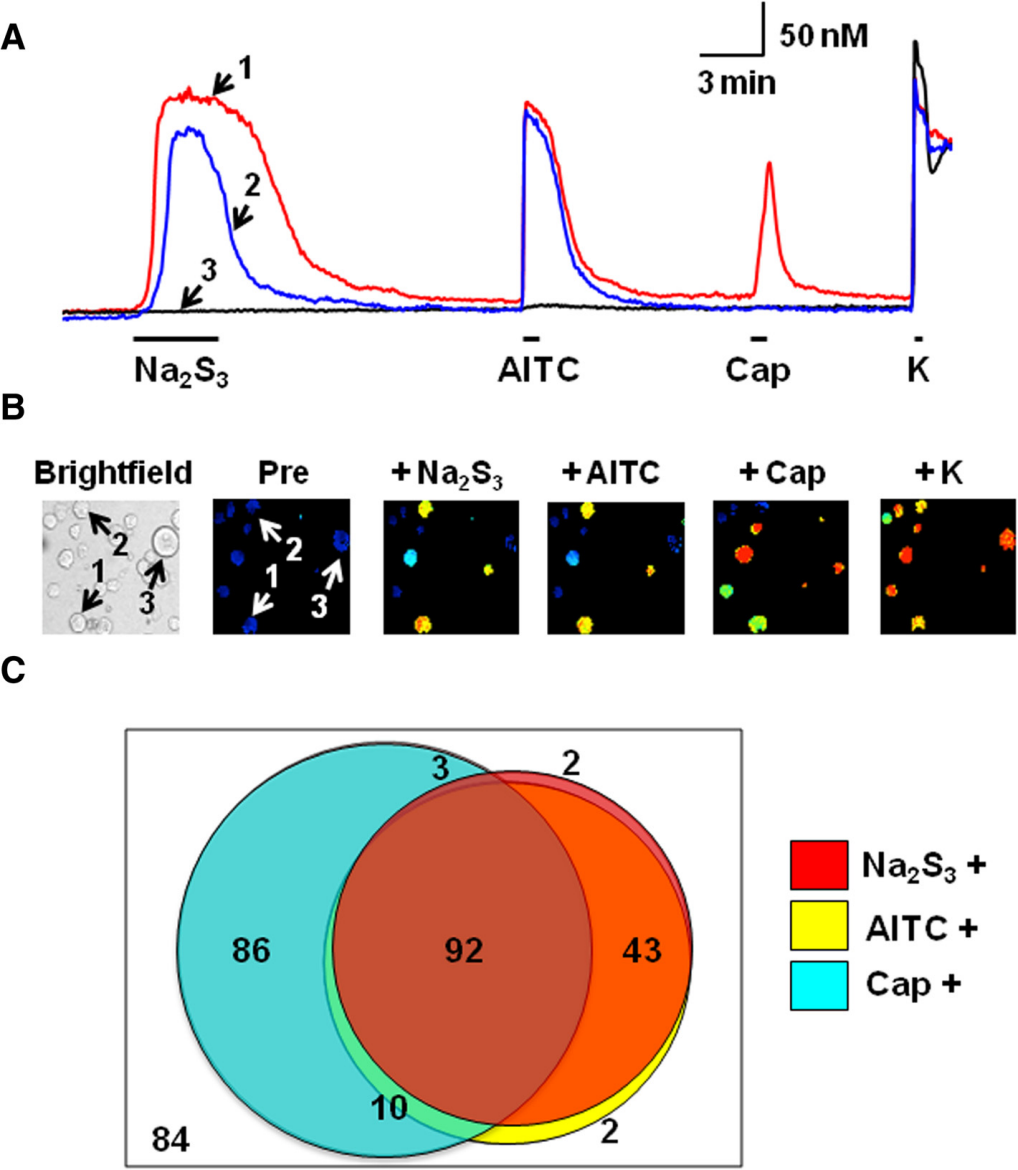
Polysulfide-responsive neurons highly correspond to TRPA1 agonist-sensitive ones. **(A)** Actual recordings of [Ca^2+^]_i_ responses to sequential application of Na_2_S_3_ (10 μM), allylisothiocyanate (AITC, 0.3 mM), capsaicin (Cap, 1 μM), and KCl (K, 80 mM). **(B)** An image under transmitted light, and pseudocolor images; before (Pre) and after the application of Na_2_S_3_ (+Na_2_S_3_), allylisothiocyanate (+AITC), capsaicin (+Cap), and KCl (+K). In a bright field image, cells with arrows (1–3) correspond to (A). **(C)** Venn diagram showing the sensitivities to Na_2_S_3_, AITC, capsaicin, and KCl (n = 322 from five mice). Numbers indicate the number of cells responding to each stimulus. A number in the outermost frame expresses the number of neurons responding to KCl alone. Note that Na_2_S_3_-responding neurons are mostly coincident with AITC-responding ones.

We examined the relationship between TRP channels and polysulfide on mouse DRG neurons. Figure 2A shows actual traces of changes in [Ca^2+^]_I_ in response to Na_2_S_3_ (10 μM) and subsequent allylisothiocyanate (AITC, a TRPA1 agonist, 0.3 mM), capsaicin (a TRPV1 agonist, 1 μM) and KCl (80 mM) of mouse DRG neurons. Most of the Na_2_S_3_-sensitive neurons were also AITC sensitive (Figure 2B and C). These data indicated that polysulfide-responding neurons highly corresponded to TRPA1 agonist sensitive-ones.

### Inhibition of polysulfide-induced [Ca^2+^]_i_ increase by TRPA1 blockers

**Figure 3:**
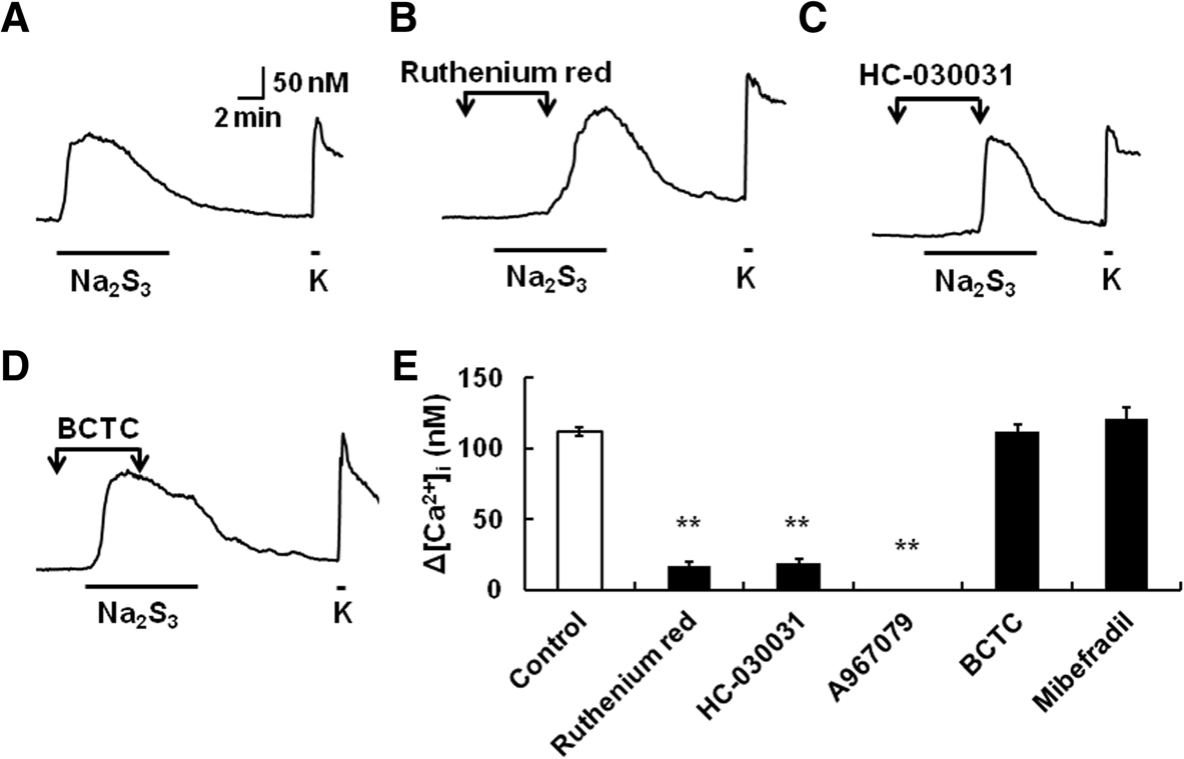
Inhibition of polysulfide-induced [Ca^2+^]_i_ increases by TRPA1 blockers. **(A)** Actual recording of [Ca^2+^]_i_ responses to Na_2_S_3_ (10 μM, 8 min) and KCl (K, 80 mM) in mouse DRG neurons. **(B-D)** The effects of ruthenium red (1 μM), HC-030031 (10 μM) and BCTC (10 μM) on the Na_2_S_3_-induced [Ca^2+^]_i_ increases. Each blocker was applied 2 min before and for 4 min during application of Na_2_S_3_. **(E)** Summarized effects of these blocking agents. Open and filled columns show the increases of [Ca^2+^]_i_ responses to Na_2_S_3_ in the absence (Control) and presence of these blocking agents, respectively. Columns with vertical lines show mean ± SEM (control; n = 201, ruthenium red; n = 32, HC-030031; n = 24, A967079 (1 μM); n = 43, BCTC; n = 43, mibefradil (10 μM); n = 44, from 3–6 mice). **P, < 0.01 vs. Control.

Next, the effects of TRP blockers on the polysulfide-induced [Ca^2+^]_i_ increases in mouse DRG neurons were examined. Figure 3 shows actual recordings of [Ca^2+^]_i_ responses to Na_2_S_3_ (10 μM) in the absence and presence of TRP blockers. Cells were stimulated with Na_2_S_3_ for 8 min and each blocker was added 2 min before and for 4 min during Na_2_S_3_ application. Ruthenium red (1 μM), a nonselective TRP channel blocker, HC-030031 (10 μM) and A967079 (1 μM), a TRPA1 blocker but not BCTC (10 μM), a TRPV1 blocker, suppressed the Na_2_S_3_-induced [Ca^2+^]_i_ increases (Figure 3B-E). It has been reported that H_2_S sensitizes T-type Ca^2+^ channels [6,7]. However, the Na_2_S_3_-evoked [Ca^2+^]_i_ increases were unaffected by mibefradil (10 μM), a T-type Ca^2+^ channel blocker. These pharmacological results suggested that TRPA1 was involved in the polysulfide-induced [Ca^2+^]_i_ increase in mouse sensory neurons.

### Absence of [Ca^2+^]_i_ responses to polysulfide in TRPA1(−/−) mouse DRG neurons

**Figure 4:**
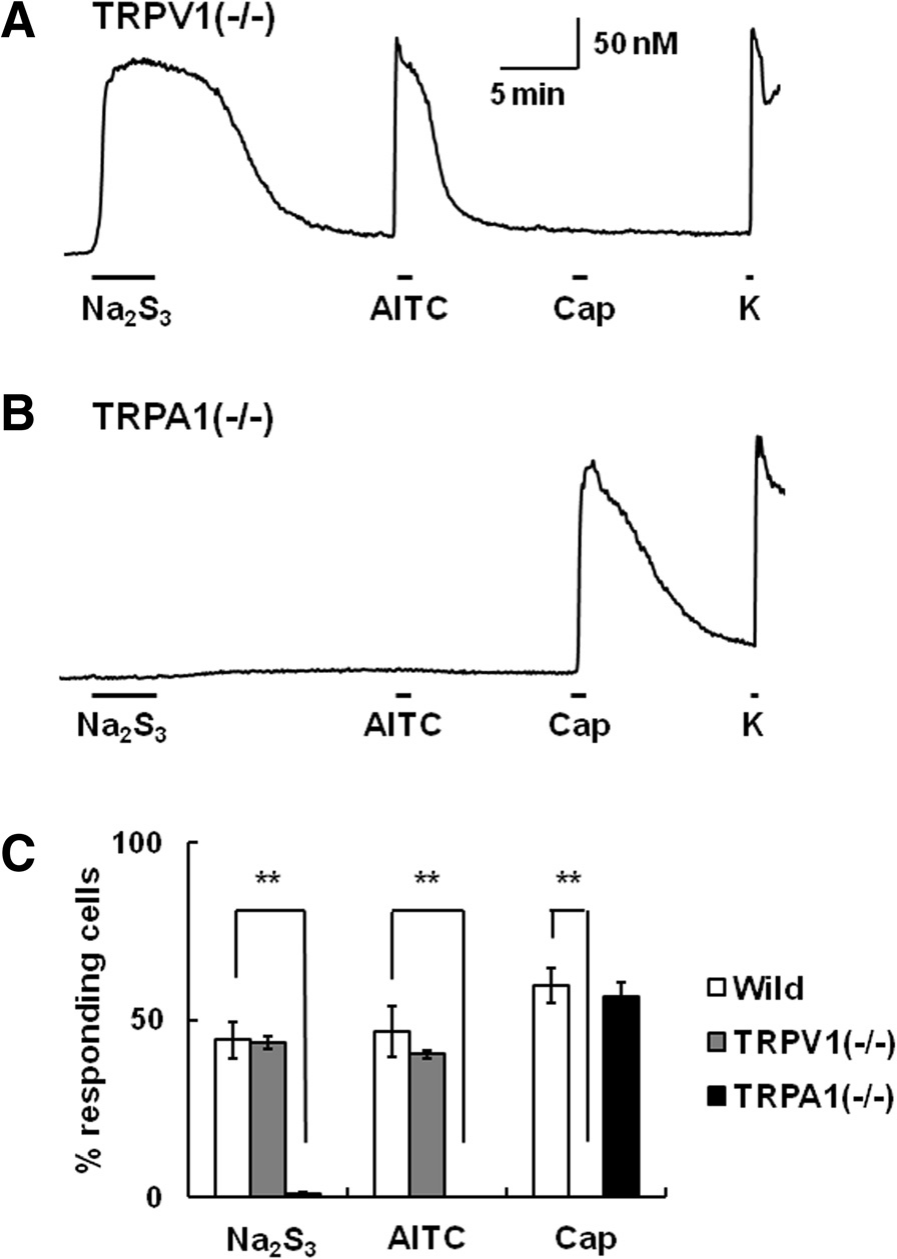
[Ca^2+^]_i_ responses to polysulfide in TRPV1(−/−) and TRPA1(−/−) mouse DRG neurons. Actual recordings of [Ca^2+^]_i_ responses to sequential application of Na_2_S_3_ (10 μM), allylisothiocyanate (AITC, 0.3 mM), capsaicin (Cap, 1 μM), and KCl (K, 80 mM) in TRPV1(−/−) **(A)** and TRPA1(−/−) **(B)** mouse DRG neurons. **(C)** Columns showing the % responding cells among all neurons in each animal. Columns with vertical lines show mean ± SEM (wild type; n = 322, TRPV1(−/−); n = 278, TRPA1(−/−); n = 253, from 3–5 mice for each genotype). **P, < 0.01.

Figure 4A and B show actual traces of [Ca^2+^]_i_ responses to Na_2_S_3_ (10 μM) and subsequent AITC, capsaicin and KCl in DRG neurons from TRPV1(−/−) and TRPA1(−/−) mice, respectively. In TRPV1(−/−) mouse DRG neurons, [Ca^2+^]_i_ responses to Na_2_S_3_ were detected in neurons that responded to AITC. Figure 4C shows the percentage of cells responding to each stimulus in wild-type, TRPV1(−/−) and TRPA1(−/−) mouse DRG neurons, indicating that the percentage of neurons responding to Na_2_S_3_ was the same in wild-type (140 of 322 cells) and TRPV1(−/−) mouse neurons (121 of 271 cells). In contrast, few cells responded to AITC or Na_2_S_3_ in DRG neurons from TRPA1(−/−) mouse (Figure 4B and C). These results clearly indicated that the polysulfide stimulated TRPA1 channels in mouse DRG neurons.

### Polysulfide causes desensitization of TRPA1 in mouse DRG neurons

**Figure 5:**
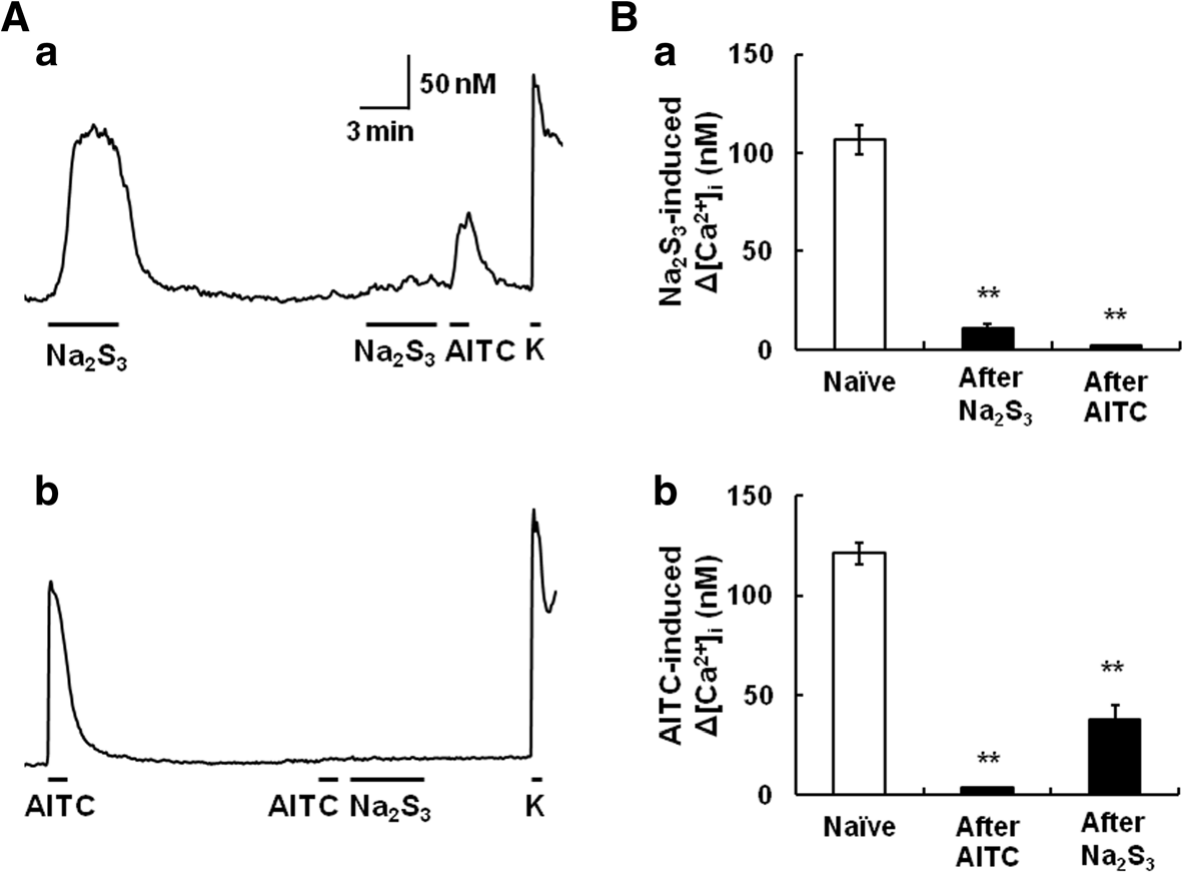
Desensitization of TRPA1 by polysulfide in mouse DRG neurons. **(A)** (a) Actual recording of [Ca^2+^]_i_ responses to Na_2_S_3_ (10 μM) twice with an interval of 15 min and then allylisothiocyanate (AITC, 0.3 mM), and KCl (K, 80 mM), and (b) those to AITC (0.3 mM) twice with an interval of 15 min and then Na_2_S_3_ (10 μM), and KCl (80 mM). **(B)** Open and filled columns show the increases of [Ca^2+^]_i_ responses to Na_2_S_3_ (a) and AITC (b) in the first stimulation (Naïve), and those in the second stimulation after each chemical, respectively. Columns with vertical lines show mean ± SEM (Ba; n = 25, Bb; n = 54). **P, < 0.01 vs. Naive.

It has been reported that AITC activates TRPA1 through covalent modification of cysteine residues and desensitizes TRPA1 [33]. We examined whether prestimulation with polysulfide influenced [Ca^2+^]_i_ responses to subsequent application of polysulfide and AITC. Figure 5A shows actual recordings of [Ca^2+^]_i_ responses to Na_2_S_3_ (10 μM) twice with an interval of 15 min and then AITC and KCl in mouse DRG neurons. We found that both [Ca^2+^]_i_ responses to Na_2_S_3_ and AITC after Na_2_S_3_ stimulation significantly decreased (Figure 5B). Similar effects were observed when AITC was applied first (Figure 5Ab and Bb). These results indicated that polysulfide desensitized TRPA1 in mouse DRG neurons.

### Polysulfide stimulates HEK 293 cells expressing mouse TRPA1 and rat TRPA1 expressing RIN-14B cells

**Figure 6:**
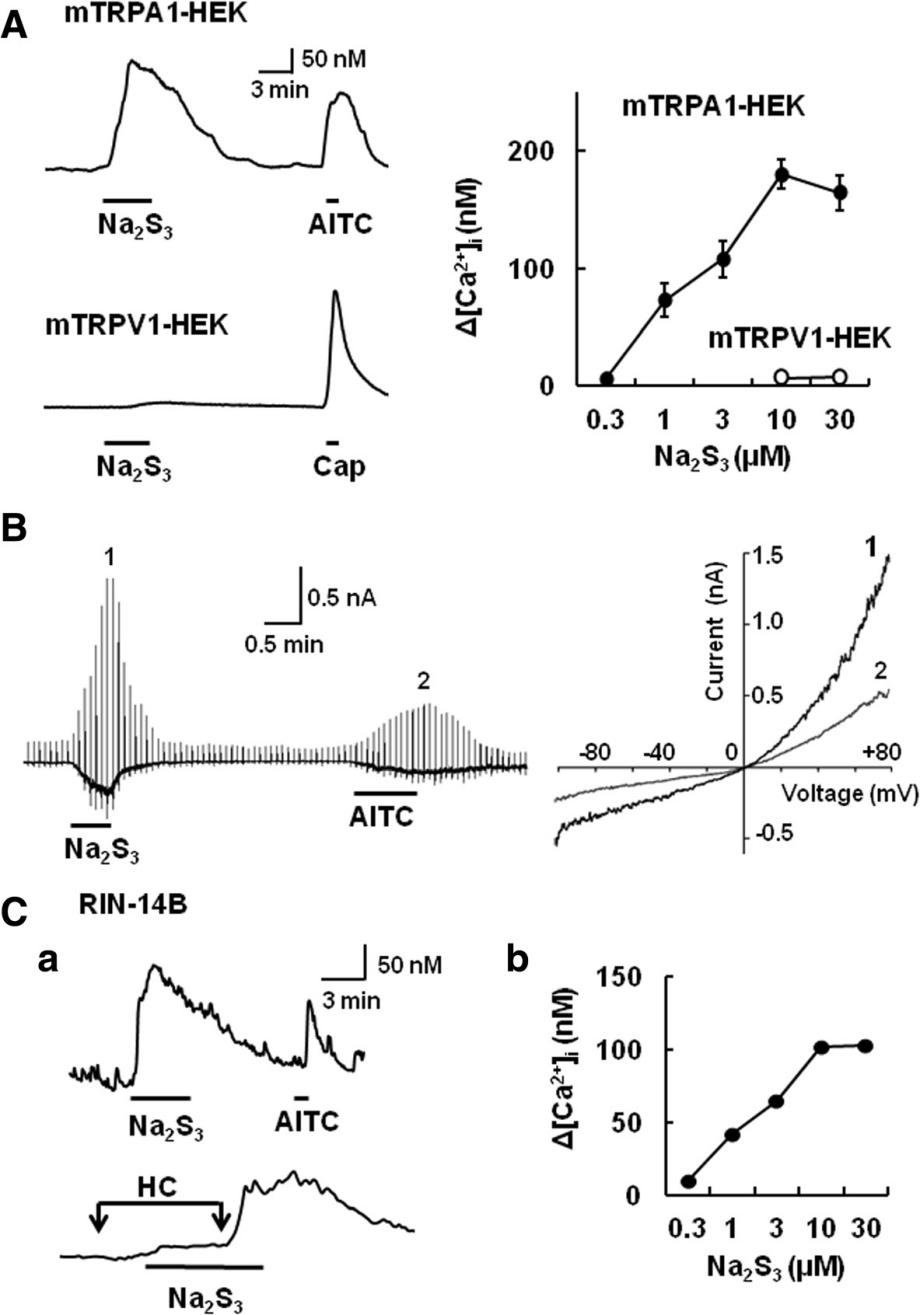
[Ca^2+^]_i_ and current responses to polysulfide in HEK 293 cells expressing mouse TRPA1. **(A)** Left shows actual traces of [Ca^2+^]_i_ responses to Na_2_S_3_ (10 μM) and allylisothiocyanate (AITC, 0.3 mM) in HEK 293 cells expressing mouse TRPA1 (mTRPA1-HEK) and those to Na_2_S_3_ and capsaicin (Cap, 1 μM) in HEK 293 cells expressing mouse TRPV1 (mTRPV1-HEK). Right graph shows that the concentration-response relationships for Na_2_S_3_ in mTRPA1-HEK (closed circles) and mTRPV1-HEK (open circles). Symbols with vertical lines show mean ± SEM (mTRPA1-HEK; n = 28-65 cells, mTRPV1-HEK; n = 52-53 cells, from three different transfections). **(B)** Representative traces of whole-cell currents activated by Na_2_S_3_ (10 μM) followed by AITC (0.3 mM) in HEK293 cells expressing mouse TRPA1. The current–voltage (I-V) curves for Na_2_S_3_ (1) and AITC (2) exhibit outward rectification. **(C,a)** An actual trace of [Ca^2+^]_i_ response to Na_2_S_3_ (10 μM) and AITC (0.3 mM) in RIN-14B cells (upper panel). The Na_2_S_3_-induced [Ca^2+^]_i_ increase is suppressed by HC030031 (10 μM, lower panel). **(C,b)** The concentration-response relationship for Na_2_S_3_ in RIN-14B cells (n = 95-150, from three experiments). Vertical lines for SEM are embedded in each symbol.

To confirm the stimulatory action of polysulfide on TRPA1, we examined its effect on HEK 293 cells expressing mouse TRPA1 (mTRPA1-HEK). As shown in Figure 6A, Na_2_S_3_ induced [Ca^2+^]_i_ increases in mTRPA1-HEK, but not HEK 293 cells expressing mouse TRPV1 (mTRPV1-HEK). The amplitude of Na_2_S_3_-induced [Ca^2+^]_i_ increase in mTRPA1-HEK increased with increasing concentrations of Na_2_S_3_ and the EC_50_ was estimated to be 3.4 ± 0.15 μM. To obtain direct evidence for TRPA1 channel activation induced by Na_2_S_3_, we performed whole-cell current recording from HEK293 cells expressing mouse TRPA1. Figure 3B shows representative current response to Na_2_S_3_ (10 μM) and the AITC (0.3 mM) in mouse TRPA1-expressing HEK293 cell. The current elicited by Na_2_S_3_ exhibited an outward rectifying current–voltage relationship similar to that evoked by AITC. In addition, we used RIN-14B, a rat enterochromaffin cell line that expresses TRPA1 constitutively [9,34]. As shown in Figure 6C, Na_2_S_3_ (10 μM) elicited [Ca^2+^]_i_ increases in RIN-14B cells. This [Ca^2+^]_i_ response was suppressed by the pretreatment with HC030031 (10 μM). The magnitude of the [Ca^2+^]_i_ increase induced by Na_2_S_3_ increased in a concentration-dependent manner (EC_50_; 3.1 ± 0.16 μM). These results indicated that polysulfide selectively stimulated TRPA1, but not TRPV1.

### N-terminal cysteine residues of TRPA1 confer sensitivity to polysulfide

**Figure 7:**
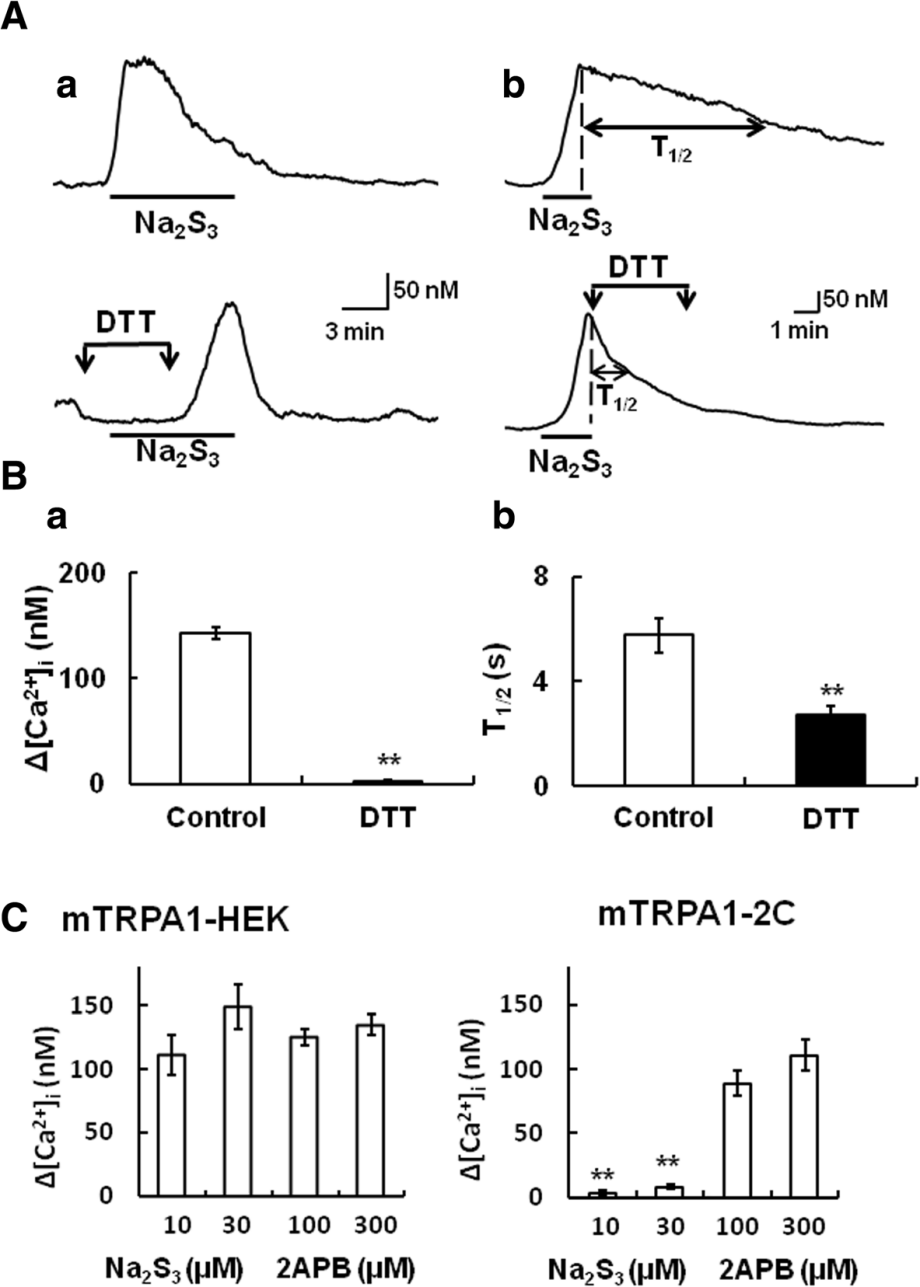
Involvement of the N-terminal cysteine residues of mouse TRPA1 in its activation by polysulfide. **(A)** The Na_2_S_3_ (10 μM)-induced [Ca^2+^]_i_ increase was inhibited by dithiothreitol (DTT) (a) 2 min before and during 4 min application of Na_2_S_3_, (b) after 4 min in HEK 293 cells expressing mouse TRPA1 (mTRPA1-HEK). The upper panels show [Ca^2+^]_i_ responses to Na_2_S_3_ without DTT, and the lower ones those in the presence of DTT. **(B)** Summarized effects of DTT. (a) Open and filled columns show the increases of [Ca^2+^]_i_ responses to Na_2_S_3_ in the absence (Control) and presence of DTT, respectively. (b) Times required for half-decline of [Ca^2+^]_i_ responses to Na_2_S_3_ (T_1/2_) in the absence (Control) and presence of DTT. T_1/2_ was calculated by subtracting the value of the time when the Na_2_S_3_-induced [Ca^2+^]_i_ increase was reduced by half from that when Na_2_S_3_-induced [Ca^2+^]_i_ increase peaked. Columns with vertical lines show mean ± SEM (a; n = 23–32, b; n = 55–63, from three different transfections). **P < 0.01. **(C)** The [Ca^2+^]_i_ increments induced by Na_2_S_3_ (10 μM and 30 μM) and 2APB (100 μM and 300 μM) in mTRPA1-HEK (left columns) and HEK293 cells expressing mouse TRPA1 mutant (mTRPA1-2C, right columns). Columns with vertical lines show mean ± SEM (wild-type mTRPA1; n = 55–72, mTRPA1-2C; n = 46–63, from three separate transfections). **P, < 0.01 vs. ∆[Ca^2+^]_i_ in mTRPA1-HEK.

It has been reported that TRPA1 is activated by reversible covalent modification of intracellular N-terminal cysteine residues in the channel [35]. We have previously reported that H_2_S modifies these cysteine residues [8]. Thus, to examine whether polysulfide activated TRPA1 by modifying cysteine residues, we tested the effects of DTT (5 mM), a reducing agent, on the polysulfide-induced [Ca^2+^]_i_ increases in mTRPA1-HEK. The [Ca^2+^]_i_ responses to Na_2_S_3_ was diminished by DTT applied before and during application of Na_2_S_3_ (Figure 7A). The increment of [Ca^2+^]_i_ evoked by Na_2_S_3_ declined faster when DTT was applied after the stimulation of Na_2_S_3_ (Figure 7B). We calculated the magnitude and the time required for the half-decline of [Ca^2+^]_i_ responses to Na_2_S_3_ to evaluate the effect of DTT.

To determine the molecular mechanism underlying the polysulfide-induced TRPA1 activation, we used a mutant mouse TRPA1 channel in which two cysteines were substituted by serines (mTRPA1-2C) [8,36]. It has been known that mTRPA1-2C loses the responsiveness to AITC, a cysteine-modifying agent but have sensitivity to 2-aminoethoxydiphenyl borate, a nonelectrophilic TRPA1 agonist [37]. We confirmed that 2APB were capable of activating this mutant channel. On the other hand, Na_2_S_3_ failed to evoke [Ca^2+^]_i_ increases in mTRPA1-2C expressing HEK 293 cells (Figure 7C). These data suggested that two N-terminal cysteine residues were essential for mouse TRPA1 activation by the polysulfide.

### Polysulfide causes acute pain in mice through TRPA1 activation

**Figure 8:**
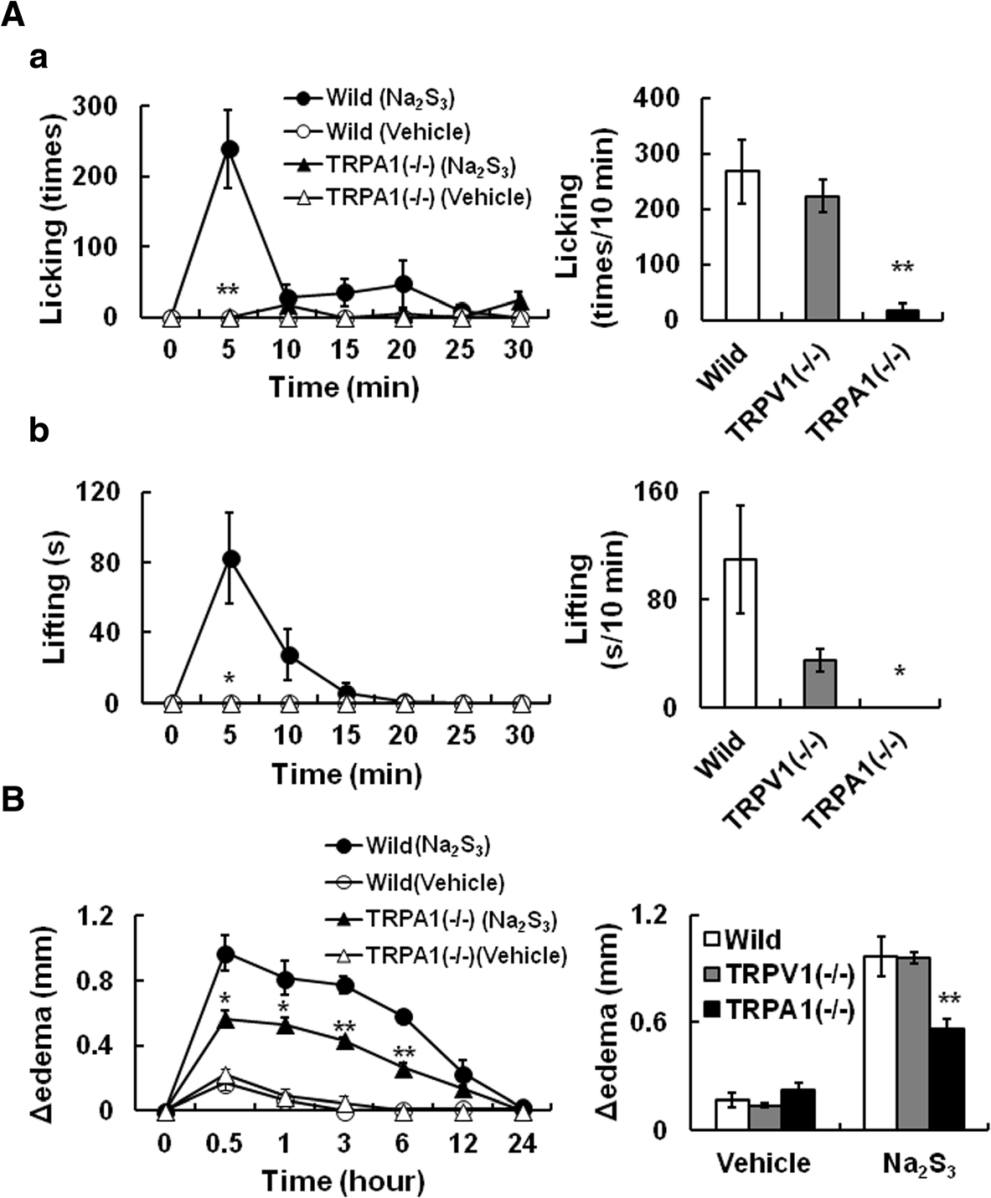
Intraplantar administration of polysulfide produces pain-related behavior in mice. **(A)** Changes in number of pain-related behaviors (a; Licking, b; Lifting) of wild-type and TRPA1(−/−) mice after intraplantarly injection of Na_2_S_3_ (500 nmol/paw) and summarized number of behaviors during 10 min after Na_2_S_3_ injection. **(B)** Left and right panel show that changes in paw thickness of wild-type and TRPA1(−/−) mice before and after intraplantarly injection of Na_2_S_3_ (left), and changes in paw thickness 30 min after injection of Na_2_S_3_ or HEPES-buffered solution (Vehicle), respectively. Symbols and columns with vertical lines show mean ± SEM (A: Wild-type; n = 5, TRPA1(−/−); n = 4, TRPV1(−/−); n = 4, B: Wild-type; n = 4, TRPA1(−/−); n = 4, TRPV1(−/−); n = 4). *P, < 0.05, **P, <0.01, vs. Wild type.

We showed that polysulfide stimulated mouse sensory neurons via the activation of TRPA1 in vitro. Since TRPA1 is a nociceptive receptor, we next investigated whether polysulfide evoked acute pain in vivo. In wild-type mice, intraplantar injection of Na_2_S_3_ induced licking and lifting of the injected paw as pain-related behaviors (Figure 8A). These nociceptive behaviors began just after the injection and almost ceased within 10 min. In a control experiment, no response was observed in mice injected with the same amount of HEPES-buffered solution as a vehicle. Similar nociceptive effects of Na_2_S_3_ were observed in TRPV1(−/−) mice. In contrast, TRPA1(−/−) mice displayed a significant attenuation of Na_2_S_3_-induced nociception. Intraplantar injection of Na_2_S_3_ also increased paw thickness (edema) in wild-type mice (Figure 8B). This Na_2_S_3_-induced edema was observed in TRPV1(−/−) mice. The extent of paw edema in TRPA1(−/−) mice was significantly less than in wild-type and TRPV1(−/−) mice. These results suggested that polysulfide caused acute pain through the activation of TRPA1 in the mice.

## Discussion

Polysulfide is a bound sulfur species derived from H_2_S. It has been reported that H_2_S stimulates a variety of ion channels such as TRPA1, TRPV1, and T-type Ca^2+^ channels [8,32,37]. Therefore, it is possible that polysulfide affects these ion channels. In the present study, we demonstrated that polysulfide activated TRPA1 based on the following evidence. First, both Na_2_S_3_ and Na_2_S_4_ stimulated only a subset of DRG neurons sensitive to AITC, a TRPA1 agonist. Second, the Na_2_S_3_-induced [Ca^2+^]_i_ increases were inhibited by ruthenium red, a nonselective TRP blocker, by HC-030031 and A967079, selective TRPA1 blockers. Third, [Ca^2+^]_i_ responses to Na_2_S_3_ were not detected in DRG neurons isolated from TRPA1(−/−) mouse. Fourth, Na_2_S_3_ elicited [Ca^2+^]_i_ and current responses in HEK 293 cells expressing mouse TRPA1. Similar to our observations, it has been reported that polysulfide elicits [Ca^2+^]_i_ increases in rat astrocytes and these responses are suppressed by ruthenium red and HC-030031 [28]. On the other hand, there are reports that H_2_S stimulates TRPV1 [37-39] and leads to neurogenic inflammation [4,5]. However, the present study showed that BCTC, a TRPV1 channel blocker, had no effect on the Na_2_S_3_-induced [Ca^2+^]_i_ increase in mouse DRG neurons. Moreover, Na_2_S_3_ was capable of eliciting [Ca^2+^]_i_ increases in TRPV1(−/−) mouse DRG neurons, and failed to stimulate HEK 293 cells expressing mouse TRPV1. Thus, we hypothesize that TRPV1 channel is not involved in the polysulfide-induced [Ca^2+^]_i_ increases in mouse DRG neurons. Since [Ca^2+^]_i_ responses to Na_2_S_3_ were not influenced by mibefradil, a T-type Ca^2+^ channel blocker, it seems unlikely that T-type Ca^2+^ channels contribute to the stimulatory action of polysulfide in mouse DRG neurons.

In the present study, some polysulfide-sensitive neurons did not show [Ca^2+^]_i_ responses to AITC (3.6% of polysulfide-sensitive neurons). When neurons were stimulated with Na_2_S_3_ twice, the magnitude of the second responses became smaller. The AITC-induced [Ca^2+^]_i_ increase after Na_2_S_3_-stimulation were also attenuated. These data suggest that polysulfide may desensitize TRPA1 resulting in AITC-insusceptibility in some neurons responding to polysulfides. Moreover, the sites of action for both chemicals are likely to be the same, as discussed below.

The TRPA1 channel is activated by covalent binding of electrophiles to internal cysteine residues [33,35]. We showed that the polysulfide-induced [Ca^2+^]_i_ increases were prevented by DTT, a reducing agent for disulfide bonds. Polysulfide contains sulfane sulfur, which releases H_2_S in the presence of DTT [40]. It may be possible that DTT reduces polysulfide to change their reactivity. Thus, DTT may influence not only the TRPA1 channel but also polysulfide itself. We found that the rate of decline of the [Ca^2+^]_i_ increment (T_1/2_) significantly decreased when DTT was applied after the washout of polysulfide, suggesting that cysteines contribute to TRPA1 channel activation by polysulfide. This idea was supported by the evidence that the polysulfide-induced TRPA1 activation disappeared in HEK 293 cells expressing cysteine mutant TRPA1. These cysteine residues are located in the N-terminal internal domain. Therefore we suggest that polysulfide produces a covalent modification of N-terminal cysteine residues for the activation of TRPA1. C422 and C634 in mouse TRPA1, being responsible for the action of polysulfide, are equivalent to C421 and C633 in human TRPA1, and these amino acids are important for sensing O_2_ [16]. It has been reported that C421 in human is also sensitive to H_2_O_2_, nitric oxide and PGJ_2_ [41]. Including the present results, several cysteine residues within the cytoplasmic N-terminal of TRPA1 channel are identified in acceptor sites for electrophilic agonists and a variety of inflammatory mediators [42].

The EC_50_ value of polysulfide was much smaller than that of H_2_S. The similar higher potency of polysulfide than H_2_S has been reported in rat astrocytes [28]. H_2_S plays a role in physiological functions through protein S-sulfhydration [2]. However, it is thought to be chemically impossible for H_2_S itself to modify proteins oxidatively. Thus, it is suspected that polysulfide acts as the intermediate species of H_2_S signaling [27]. The H_2_S level of the polysulfide (10 μM)-containing solution, the concentration that induced nearly the maximal [Ca^2+^]_i_ increment, was estimated to be 0.4 μM or less. Since the EC_50_ of H_2_S for TRPA1 activation is reported to be 36.0 ± 2.5 μM in HEK 293 cells expressing mouse TRPA1 [8], indirectly produced H_2_S may have little involvement in the polysulfide-induced [Ca^2+^]_i_ increases. In other words, polysulfide itself could activate TRPA1 channels rather than through H_2_S production. It has been reported that polysulfide causes protein S-sulfhydration, that is, conversion of cysteinyl thiolates (Cys-S^−^) to persulfides (Cys-S-S^−^) [27]. NMDA receptor activity may be enhanced by polysulfide via S-sulfhydration [25]. This may also be the case for TRPA1 activation by polysulfide, which may add bound sulfane sulfur of cysteine residues of the channel.

It is known that H_2_S is involved in nociception and hyperalgesia [8,43-46]. The present results clearly showed that acute pain and tissue edema were induced by intraplantar injection of polysulfide in wild-type and TRPV1(−/−) mice. These effects of polysulfide were small in TRPA1(−/−) mice. It has been reported that TRPA1 is involved in neuropathic, inflammatory pain and edema [47-49]. Although these reports support the involvement of TRPA1 in nociception, mechanisms of agonist-induced edema formation are not simple. AITC evokes edema which is completely inhibited by TRPA1 antagonist [47] and the edema induced by lipopolysaccharide is not observed in TRPA1(−/−) mice [48]. However, there is a report that AITC-induced edema is still observed in TRPA1-deficient mice [50]. Moreover, 4-oxo-2-nonenal-induced edema formation is not affected by deletion of TRPA1-gene and TRPA1 antagonist [51]. In the present study, polysulfide-induced edema was decreased but not abolished in TRPA1(−/−) mice. These differences might depend on TRPA1 agonist used and/or experimental conditions. Nevertheless, our data suggest that polysulfide activates the TRPA1 channel and then might elicit neurogenic inflammation. The H_2_S level in serum rises in inflammation via upregulation of H_2_S-producing enzymes [52,53]. There is a possibility that H_2_S generated under the inflammatory condition may form polysulfide, which activates nociceptive TRPA1. Since putative parental H_2_S is reported to be increased under inflammatory conditions, it is important to estimate endogenous polysulfide levels in relation to any inflammatory conditions. These works remained to be performed in the future. PGJ_2_ and protons are known to be endogenous agonists for the TRPA1 channel [54,55]. Since these TRPA1 ligands are able to induce nociception in vivo, it may be possible that polysulfide also acts as an endogenous ligand for the nociceptive TRPA1 channel.

## Conclusions

The present study demonstrates that polysulfide is more potent TRPA1 agonist than parental H_2_S. Polysulfide is known to promote protein sulfhydration more efficiently than H_2_S [25]. Some conditions are known to be associated with sulfhydration, including Parkinson disease and ischemia reperfusion injury [3,56]. However, the mechanisms of production, storage, and the stimulation that facilitates polysulfide-release remain to be clarified [24]. Further study will enhance the potential therapeutic value of polysulfide.

## Methods

All protocols for experiments on animals were approved by the Committee on Animal Experimentation of Tottori University. All efforts were made to minimize the number of animals used.

### Isolation and culture of mouse DRG neurons

We used adult mice of either sex (4–8 weeks). C57BL/6 mice, TRPA1(−/−) mice (kindly provided by Dr. D. Julius, University of California), and TRPV1(−/−) mice (The Jackson Laboratory, BarHarbor, ME, USA) were euthanized by inhalation of CO_2_ gas. All efforts were made to minimize the number of animals used.

Mouse DRG cells were isolated and cultured as described previously [8]. In brief, DRG cells were removed and dissected in phosphate-buffered saline (PBS: in mM, 137 NaCl, 10 Na_2_HPO_4_, 1.8 KH_2_PO_4_, 2.7 KCl) supplemented with 100 U/ml penicillin G and 100 μg/ml streptomycin. Then the isolated ganglia were enzymatically digested for 30 min at 37°C in PBS-containing collagenase (1 mg/ml, type II, Worthington, Lakewood, NJ, USA) and DNase I (1 mg/ml, Roche Molecular Biochemicals, Indianapolis, IN, USA). Subsequently, the ganglia were immersed in PBS-containing trypsin (10 mg/ml, Sigma, St. Louis, MO, USA) and DNase I (1 mg/ml) for 15 min at 37°C. After enzyme digestion, the ganglia were washed with the culture medium, Dulbecco’s-modified Eagle’s medium (DMEM, Sigma) supplemented with 10% fetal bovine serum (Sigma), penicillin G (100 U/ml) and streptomycin (100 μg/ml). DRG cells were obtained by gentle trituration with a fine-polished Pasteur pipette. Then the cell suspension was centrifuged (800 rpm, 2 min, 4°C) and the pellet-containing cells were resuspended with the culture medium. Aliquots were placed onto glass cover slips coated with poly-DL-lysine (Sigma) and cultured in a humidified atmosphere of 95% air and 5% CO_2_ at 37°C. In the experiment, cells cultured within 24 h were used.

### Heterologous expression in HEK 293 cells

Cells were transfected using 1 μg of mouse TRPA1 (mTRPA1), mouse TRPV1 (mTRPV1) and a double cysteine mutant of mTRPA1 (C422S/C634S, mTRPA1-2C) [36]. Human embryonic kidney (HEK) 293 cells were cultured in DMEM supplemented with 10% FBS, 100 U/ml penicillin G and 100 μg/ml streptomycin. Cells were transfected with the expression vectors using a transfection reagent (Lipofectamine 2000, Invitrogen) and used 24 h after transfection.

### Culture of RIN-14B cells

The RIN-14B cells were purchased from DS Pharma Biomedical Co., Ltd. (Osaka, Japan). Cells were cultured in RPMI1640 medium (Wako) supplemented with 10% FBS, 100 U/ml penicillin G and 100 μg/ml streptomycin.

### Measurement of [Ca^2+^]_i_

The intracellular Ca^2+^ concentrations ([Ca^2+^]_i_) in individual cells were measured with the fluorescent Ca^2+^ indicator fura-2 by dual excitation using a fluorescent-imaging system controlling illumination and acquisition (Aqua Cosmos, Hamamatsu Photonics, Hamamatsu, Japan) as described previously [57]. To load fura-2, cells were incubated for 40 min at 37°C with 10 μM fura-2 AM (Molecular Probes) in HEPES-buffered solution (in mM: 134 NaCl, 6 KCl, 1.2 MgCl_2_, 2.5 CaCl_2_, 5 glucose, and 10 HEPES, pH 7.4). A coverslip with fura-2-loaded cells was placed in an experimental chamber mounted on the stage of an inverted microscope (Olympus IX71) equipped with an image acquisition and analysis system. Cells were illuminated every 5 s with lights at 340 and 380 nm, and the respective fluorescence signals at 500 nm were detected. The fluorescence emitted was projected onto a charge-coupled device camera (ORCA-ER, Hamamatsu Photonics) and the ratios of fluorescent signals (F_340_/F_380_) for [Ca^2+^]_i_ were stored on the hard disk of a computer. Cells were continuously superfused with the external solution at a flow rate of ∼ 2 ml/min. The composition of high-KCl solution was (in mM) 80 KCl, 60 NaCl, 1.2 MgCl_2_, 2.5 CaCl_2_, and 10 HEPES (pH 7.4 with NaOH). All experiments were carried out at room temperature (22–25°C).

### Immunocytochemistry

After the measurement of [Ca^2+^]_i_ in cultured cells, cells were fixed with 4% paraformaldehyde and then immunostained with a rabbit antiserum to protein gene product 9.5 (PGP9.5, diluted 1:5000, Chemicon, Temecula, CA, USA) as the 1st antibody. Subsequently this antibody was visualized with Alexa-labeled goat anti-rabbit IgG (10 μg/ml, Invitrogen) as the 2nd antibody. A mounting agent including Hoechest 33752 was used for nuclear staining.

### Whole-cell current recording

HEK293 cells expressing mouse TRPA1 were mounted in an experimental chamber and superfused with HEPES-buffered solution as for Ca imaging experiments. The pipette solution contained (in mM: 140 KCl, 10 HEPES, 5 EGTA, pH 7.2 with KOH). The resistance of patch electrodes ranged from 4 to 5 MΩ. The whole-cell currents were sampled at 5 kHz and filtered at 1 kHz using a patch-clamp amplifier (Axopatch 200B; Molecular Devices, Sunnyvale, CA) in conjunction with an A/D converter (Digidata 1322A; Molecular Devices). Membrane potential was clamped at −60 mV and voltage ramp pulses from −100 mV to +80 mV for 100 ms were applied every 5 s.

### Measurement of H_2_S

The H_2_S concentration in polysulfide-containing HEPES-buffered solution was measured according to a protocol described previously [9]. In brief, Na_2_S_3_ (10 μM)-containing HEPES-buffered solution (0.5 ml) was added to 10% trichloroacetic acid (0.25 ml), 1% zinc acetate (0.25 ml). The solutions were mixed with 20 mM N,N-dimethyl-p-phenylenediamine in 7.2 M HCl (133 μl) and 30 mM FeCl_3_ in 1.2 M HCl (133 μl) and incubated for 10 min at room temperature. Then, the absorbance at 670 nm was measured and the H_2_S concentration of each sample was calculated from the calibration data.

### Behavioral experiments

Mice were placed in cages for 30 min before experiments. Twenty microliters of the HEPES-buffered solution (vehicle), which was similar in composition to that used in in vitro experiments, was first injected intraplantarly into the left hind paw as a control. The number of times each mouse licked the injected paw and the time of lifting it were counted for 30 min after the injection. Subsequently, the same amount of Na_2_S_3_ (500 nmol/paw) was injected into the right hind paw, and the number and time of pain-related behaviors were counted for 30 min. To assess the development of edema, paw thickness was measured with a digital micrometer (AS ONE, Osaka) before and at several time points (0.5, 1, 3, 6, 12, 24 h) post injection. The results are expressed as paw thickness variation (Δedema, in millimeters), calculated by subtracting the value obtained at each time point posttreatment from that obtained before treatment.

### Chemicals

The following drugs were used (vehicle and concentration for stock solution). Allylisothiocyanate (AITC, DMSO, 1 M) was from Nakarai, Tokyo, Japan. 2-Aminoethoxydiphenyl borate (2APB, dimethyl sulfoxide (DMSO), 1 M), capsaicin (ethanol, 1 mM), cremophor EL (distilled water: DW, 1%), HC-030031 (DMSO, 0.1 M), and mibefradil (DW, 0.05 M) were obtained from Sigma. A967079 (DMSO, 0.01 M) was from Focus Biomolecules (Pennsylvania, USA). N-(4-t-butylphenyl)-4-(3-chloropyridin-2-yl) tetrahydropyrazine-1(2H)-carboxamide (BCTC, DMSO, 0.05 M) was from BIOMOL Research Laboratories, Inc., Plymouth Meeting, PA, USA. Dithiothreitol (DTT, DW, 1 M), polysulfides (Na_2_S_3_ and Na_2_S_4_), and ruthenium red (DW, 0.01 M) were from Wako, Osaka, Japan. Polysulfide-containing aqueous solution was made just before each experiment. All other drugs used were of analytical grade.

### Data analysis

The data are presented as the mean ± SEM (n = number of cells). For comparison of two groups, data were analyzed by the unpaired Student’s t test, and for multiple comparisons, one-way ANOVA following by the Tukey-Kramer test was used. Differences with a P-value of less than 0.05 were considered significant. Values of the 50% maximal effective concentrations (EC_50_) were determined using Origin software 9.1 J (Origin-Lab). The average percentage (±SEM) of polysulfide-responsive cells was calculated from the percentage obtained with each cover glass.

## Abbreviations

AITC: Allyl isothiocyanate
2APB: 2-aminoethoxydiphenyl borate
DRG: Dorsal root ganglia
DMSO: Dimethylsulfoxide
HEK: Human embryonic kidney
[Ca^2+^]_i_: Intracellular Ca^2+^ concentration
PGP9.5: Protein gene product 9.5
TRPA1: Transient receptor potential ankyrin 1
TRPV1: Transient receptor potential vanilloid 1
